# Severe immediate hypersensitivity to gadolinium contrast agent after targeted treatment in a patient with alveolar soft part sarcoma: A case report and review of literature

**DOI:** 10.1097/MD.0000000000036092

**Published:** 2023-11-17

**Authors:** Hongxia Yan, Jianxin Zhang, Xinfeng Cai, Zhiying Hao, Zhe Guan

**Affiliations:** a Department of Pharmacy, Shanxi Province Cancer Hospital/Shanxi Hospital Affiliated to Cancer Hospital, Chinese Academy of Medical Sciences/Cancer Hospital Affiliated to Shanxi Medical University, Taiyuan, China; b Department of MRI/CT, Shanxi Province Cancer Hospital/Shanxi Hospital Affiliated to Cancer Hospital, Chinese Academy of Medical Sciences/Cancer Hospital Affiliated to Shanxi Medical University, Taiyuan, China; c Department of Bone and Soft Tissue Oncology, Shanxi Province Cancer Hospital/Shanxi Hospital Affiliated to Cancer Hospital, Chinese Academy of Medical Sciences/Cancer Hospital Affiliated to Shanxi Medical University, Taiyuan, China.

**Keywords:** alveolar soft part sarcoma, gadolinium-based contrast agents (GBCAs), hypersensitivity, immune response, targeted therapy

## Abstract

**Rationale:**

Gadolinium-based contrast agents (GBCAs), benefiting from good tolerance and safety, become the priority contrast agents in magnetic resonance imaging. Serious hypersensitivity reactions caused by GBCAs are rare, but occur occasionally. The “immune surveillance” theory proposes that lowered immune function exists in patients with malignance, which decrease the occurrence of atopy. Natural immunosurveillance that enhanced by effective treatment of malignance may increase the risk of hypersensitivity.

**Patient concerns:**

A 29-year-old female patient suffering from intensive pain with left leg mass was admitted in our hospital.

**Diagnoses:**

The patient was diagnosed with alveolar soft part sarcoma by histopathology and revealed destruction of the left fibula and lung metastasis by computed tomography scan, and treated with anlotinib hydrochloride, a multi-targeted tyrosine kinase inhibitor. After 4 cycles of effective targeted therapy, the patient developed severe immediate hypersensitivity due to gadopentetate dimeglumine-enhanced magnetic resonance imaging.

**Interventions and outcomes:**

The vital signs of the patient returned to normal after rescue. Since then, the patient has not used gadolinium contrast agent again, and currently the condition is stable and still alive.

**Lessons:**

Severe immediate hypersensitivity might be occurred by gadolinium contrast agent in patients with malignance after effective treatment. We explored the potential mechanism of GBCA-inducing hypersensitivity in detail, by especially focusing on the changes of immune environment. Furthermore, we propose new ideas for the safe use of GBCAs in patients with malignancies.

## 1. Introduction

Contrast-enhanced magnetic resonance imaging (MRI) provides the necessary imaging evidence for the clinical diagnosis and curative effect evaluation of malignant tumors. Since the 1980s, gadolinium-based contrast agents (GBCAs) have been the main pillar in clinical diagnosis as they enhanced the resolution of MRI, and improved the detection rates of micro lesions, which show good tolerance and safety.^[1]^ Adverse reactions related to GBCAs have been gradually reported^[2–4]^ with the increase of their application. Though most of the GBCAs’ adverse effects were mild, more serious or even lethal events should attract more attention to ensure their safety usage. Patients with malignancies are more frequently exposed to GBCAs for periodic assessment of therapeutic regimen. Immunodeficiency is common in patients with malignancies, which is associated with generally low incidence rates of hypersensitivity.^[5]^ However, in this case, the serious immediate hypersensitivity caused by gadopentetate dimeglumine (Gd-DTPA), which is the first linear ionic GBCA for enhanced MRI, had not been avoided.

The purpose of our work was to report an extremely rare case of hypersensitivity induced by GBCAs. In addition, we not only analyzed the possible mechanisms of inducing severe immediate hypersensitivity and the corresponding countermeasures, but also proposed a new viewpoint for the clinical management of GBCAs in patients with malignant tumors.

## 2. Case presentation

**Figure 1.**
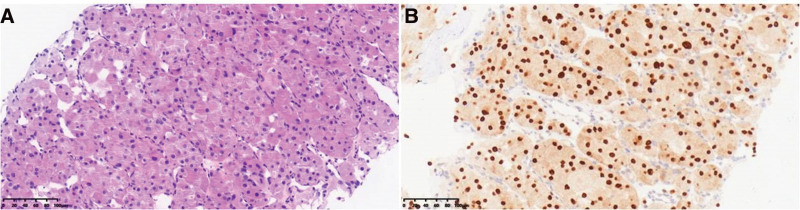
Pathological images of the patient. (A) Hematoxylin and eosin staining. (B) Immunohistochemical staining.

A 29-year-old female patient (height, 173 cm; weight, 63 kg) suffering intensive pain with left leg mass for >1 month was admitted in our hospital. She was diagnosed with alveolar soft part sarcoma by histopathology (Fig. 1), and computed tomography scan showed destruction of the left fibula and lung metastasis. She denied any history of food and drug allergies or asthma. She was treated with anlotinib hydrochloride, a multi-targeted tyrosine kinase inhibitor on July 20, 2020. The patient underwent contrast-enhanced MRI of the left tibiofibular bone using 20 mL gadodiamide (Gd-DTPA-BMA) on July 14 and September 8, 2020. To evaluate the therapeutic effect of 4 cycles of anlotinib hydrochloride, 15 mL Gd-DTPA (Magnevist, Bayer, Batch number KT05TTJ) were used for the left leg MRI enhanced scan on November 12, 2020. Approximately 2 minutes after the Gd-DTPA injection, the patient raised her hand to indicate discomfort, presented with nausea and vomiting, and a pale face. The MRI medical staff stopped the machine urgently and transferred the patient to a stretcher for electrocardiogram, blood oxygen saturation, and blood pressure monitoring. At this time, the patient’s blood pressure and heart rate could not be measured and she could not respond to inquiries. The medical staff immediately established the venous pathway and administered oxygen therapy. In addition, epinephrine (0.5 mg, 1 g/1000 mL) was administrated intramuscularly, methylprednisolone (40 mg) was injected intravenously, and diphenhydramine (20 mg) was injected intramuscularly. Ten minutes later, she gradually developed spontaneous breathing and consciousness. Physical examination revealed a blood pressure of 48/29 mm Hg and a heart rate of 135 bpm. The patient was administered a 200 mg intravenous drip of dopamine and a 500 mL hydroxyethyl starch injection for volume expansion. After 10 minutes, the patient’s blood pressure was 80/50 mm Hg. The patient was conscious and was transferred to intensive care unit for further treatment. After 12 hours of acid suppression and maintenance of water and electrolyte balance in the intensive care unit, the patient’s vital signs were stable the next morning, and she was transferred to the general ward to continue primary treatment. After 3 days of primary treatment, the patient was discharged.

## 3. Discussion

The case was a severe immediate hypersensitivity reaction that occurred in a patient with a malignant tumor when she underwent enhanced MRI to evaluate the efficacy of 4 cycles of anlotinib hydrochloride. The patient was diagnosed with anaphylactoid shock induced by Gd-DTPA given the time relationship between symptom onset and injection, and rescued in time. Of note, the patient did not have any adverse reactions after using Gd-DTPA-BMA twice before, but there was a serious immediate hypersensitivity reaction with Gd-DTPA during the third MRI. We explored the potential mechanisms from patients and drugs to disease.

First, previous studies have pointed out that patients’ sex (woman), age (18–65 years), MRI site (abdomen and/or pelvis), and allergy history (including rhinitis or asthma, any food allergy, and allergies to other drugs) are associated with an increased risk of serious reactions to GBCAs.^[2–4]^ In particular, the incidence of instant hypersensitivity in patients increased with an increase in exposure time to MR contrast agents. In this case, the patient had no history of food or drug allergies and had been exposed to GBCA twice before, without any problems. Therefore, age, sex, and previous repeated usage of GBCA played an important role in the severe immediate hypersensitivity induced by GBCAs.

Second, the chemical structure of a drug is related to its hypersensitivity reactions. GBCAs are divided into ionic and nonionic types, such as Gd-DTPA and Gd-DTPA-BMA, according to their net charge. Raisch *et al* suggested that Gd-DTPA caused more anaphylactic shock than Gd-DTPA-BMA.^[6]^ Gd-DTPA is characterized by the presence of ionic bonds in the chemical structure of molecules and the existence of free ion residues in solution, which increases the toxicity of drug ecological agents and reduces their biological tolerance.^[7]^ In contrast, due to the existence of ionic bonds, Gd-DTPA has a higher osmotic pressure than Gd-DTPA-BMA. Hyperosmolality-related direct membrane effects, complement activation, and immunoglobulin E (IgE)-mediated effects can lead to hypersensitivity. Furthermore, these molecules are classified based on their molecular structure (being linear or macrocyclic) as well as their net charge. Macrocyclic chelation ligands are more stable, which may be relevant for toxicity. Some researchers believe that the direct effect on basophils or mast cell membranes is related to the chemical structure or osmotic pressure of the contrast medium.^[8]^ Structural features and osmolality of various GBCAs used in clinic were displayed in Table 1. With regard to hypersensitivity induced by GBCAs, Galera *et al*^[9]^ reported that hyperosmolar GBCAs might be IgE mediated rather than nonspecific histamine release, and the more serious the reaction was, the more likely it was to involve the IgE-mediated mechanism.

**Table 1:** Structural features and osmolality of gadolinium-based contrast agents.

Contrast agent	Ligand class	Ionic	Osmolality (mOsmol/kg)
Gadodiamide	Linear	Nonionic	780
Gadoversetamide	Linear	Nonionic	1110
Gadopentetate dimeglumine	Linear	Ionic	1960
Gadobenate dimeglubine	Linear	Ionic	1970
Gadoxetic acid disodium	Linear	Ionic	688
Gadofosveset trisodium	Linear	Ionic	825
Gadobutrol	Macrocycle	Nonionic	1603
Gadoteridol	Macrocycle	Nonionic	630
Gadoterate	Macrocycle	Ionic	1350

**Figure 2.**
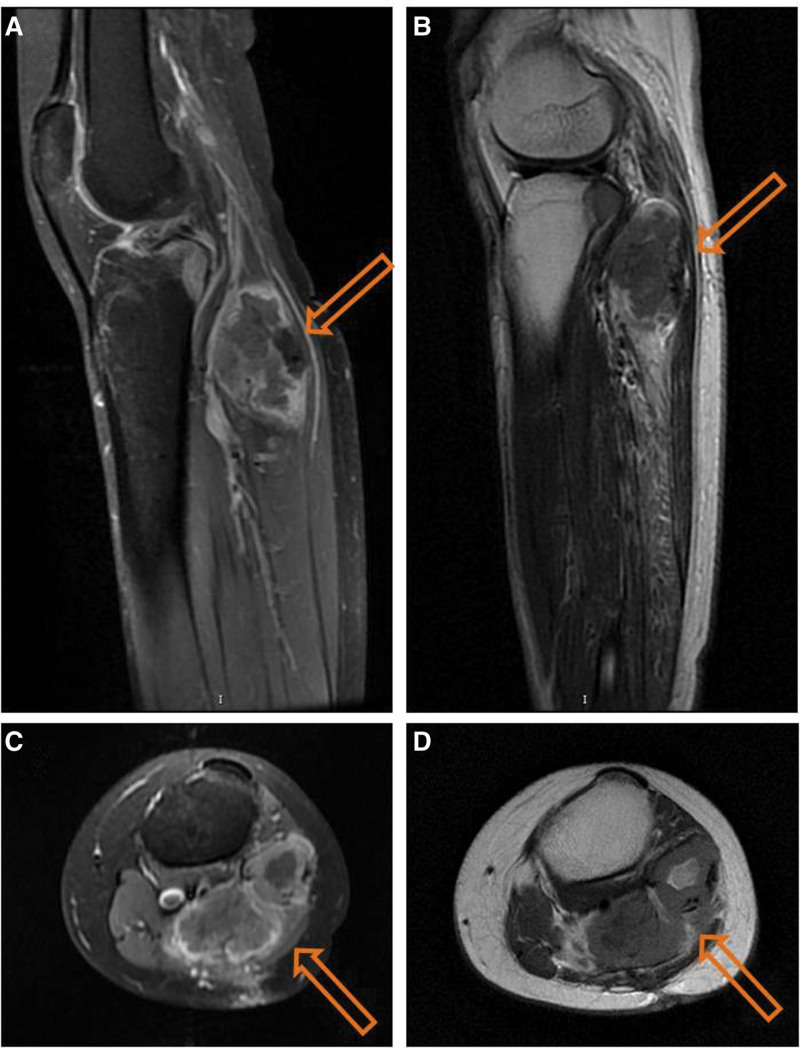
MRI of left leg mass. Sagittal image: (A) before targeted therapy (58 mm × 39 mm); (B) after 4 cycles of anlotinib hydrochloride therapy (53 mm × 35 mm). Axial image: (C) before targeted therapy (34 mm × 65 mm); (D) after 4 cycles of anlotinib hydrochloride (30 mm × 59 mm) (response evaluation criteria in solid tumors as stable disease).

Finally, we speculate that a hypersensitivity reaction was related to the natural immunosurveillance that enhanced by effective treatment of malignance. The “immune surveillance” theory proposes that enhanced immune responsiveness may be provoked atopy, which defined by exaggerated IgE responses. There is an inverse association between IgE sensitization and the risk of malignant tumor.^[10–12]^ The IgE levels of patients with malignant tumors are often much lower than those of the general population, less susceptible aroused hypersensitivity.^[5]^ Supporting evidence shows that solid tumors are most common in IgE-deficient patients.^[13]^ Several cancer therapeutic regimens, such as chemotherapy, radiotherapy, and targeted therapy can increase the concentration of IgE.^[14–16]^ High IgE levels may reflect the natural activation of immune monitoring mechanisms. Early studies showed an increased risk of IgE-mediated type I hypersensitivity in some patients with head and neck cancer after targeted drug therapy.^[15]^ The patient was diagnosed with alveolar soft part sarcoma and received 4 targeted therapy sessions with arotinib hydrochloride. Alveolar soft part sarcoma that shows insensitivity to radiotherapy and chemotherapy, is a rare soft tissue sarcoma with high risk of lung and brain metastases. Targeted drugs are more effective for it. Arotinib hydrochloride is a multi-target tyrosine kinase inhibitor that inhibits tumor proliferation and angiogenesis. When the malignant tumor was effectively controlled with targeted therapy (Fig. 2), immune monitoring might be activated, and IgE level was elevated, which increased the risk of severe hypersensitivity. Unfortunately, the IgE was not collected during therapy in this report because it was not awarded a priori that natural immunosurveillance would be enhanced by effective treatment of malignance. Henceforth, the patient still received targeted therapy, but no gadolinium contrast agent was used again.

According to the literatures of severe immediate hypersensitivity due to gadolinium contrast agent in patients with malignances, only 9 reports were found^[17–25]^ summarized in Table 2. The treatment of malignances was not mentioned in literatures. Patients who experienced severe hypersensitivity with Gd-DTPA also had a higher incidence of prior allergic events, with 3 of 4 patients having a history of allergy to something. Severe immediate hypersensitivity was caused by macrocyclic GBCAs in the remaining 5 reports. These cases suggest that we should pay more attention to patients with malignant tumors, even if they did not have any discomfort in previous usage of GBCAs.

**Table 2:** Summary of reports of severe immediate hypersensitivity to gadolinium contrast agent in the patient with malignance.

Author, year	GBCAs	Gender	Age	Diagnosis	History of allergy
Nomura M, et al, 1993^[17]^	Gd-DTPA	Female	24	Suprasellar brain tumor	Rhinallergosis
Katoh A, et al, 1993^[18]^	Gd-DTPA	Female	18	Tuberous sclerosis and renal tumors	Asthma bronchiale
Jordan RM, et al, 1995^[19]^	Gd-DTPA	Female	67	Suprasellar mass	A history of severe chronic obstructive pulmonary disease
Li A, et al, 2006^[20]^	Gd-DTPA	Not described	77	Malignant melanoma of the nostril	No
Simons CW, et al, 2009^[21]^	Gadoteridol	Female	58	Breast cancer and hypertension	No
Kerget B. 2018^[22]^	Gadobutrol	Female	46	Submandibular mass	No
Moreno Escobosa MC, et al, 2018^[23]^	Gadobutrol	Male	45	Astrocytoma	Not described
Sellaturay, P, et al, 2018^[24]^	Gadobutrol	Male	47	Low-grade glioma	No
Malone J, et al, 2020^[25]^	Gadobutrol	Female	46	Hemangiopericytoma	Allergic to penicillin, sulpha drugs, and dimenhydrinate

GBCAs = gadolinium-based contrast agents, Gd-DTPA = gadopentetate dimeglumine.

However, there is no clear prophylactic management for serious immediate hypersensitivity reactions caused by gadolinium contrast agents. There are different preventive measures for patients with a history of allergies. First, the American Society of Radiology recommends the use of antihistamines and corticosteroids as premedications. At the same time, it also pointed out that premedication might cause breakthrough reactions, which also caused allergic reactions.^[1]^ Second, similar to iodine contrast agents, the European Academy of Allergy Asthma and Clinical Immunology Guidelines propose that patients with an allergic history should undergo skin tests before using contrast agents. Allergic reactions induced by contrast agents were evaluated by an allergy expert group, who performed skin tests to determine the varieties that might cause allergic reactions and identify alternative contrast agents.^[26–28]^ Third, some researchers have suggested general anesthesia to prevent hypersensitivity in patients with an allergic history. General anesthesia inhibits consciousness-dependent reactions, such as anxiety and autism phobia, but does not suppress the release of basophils and mast cells induced by iodide or GBCAs, and the subsequent immediate hypersensitivity.^[29]^

Nevertheless, these measures have their own benefits and drawbacks. We try to find a method for the safe use of GBCAs in patients with malignant tumors, which is more frequently exposed to GBCAs and has its own particularity. The immune function of cancer patients is low. After treatment, it may increase the concentration of IgE and the risk of its mediated hypersensitivity. Therefore, we suggest that IgE should be detected regularly during cancer treatment. Before using GBCAs, the risk of allergic reactions can be judged by comparing the IgE levels in the last 2 times, and medical staff should also take measures against rare reactions. Moreover, a comparison of IgE expression differences can also be used to infer the safe use of other drugs that induce hypersensitivity to IgE.

## 4. Conclusions

In conclusion, although GBCAs show good safety and tolerance, more attention should be paid to the occurrence of hypersensitivity in patients with malignancy. We analyzed the reasons for hypersensitivity, including age, sex, previous repeated use, ionic and high osmotic GBCA, and changes in IgE levels. It has been proposed that IgE is an important indicator for evaluating the hypersensitivity reaction of patients with malignant tumors during treatment, which enriches the clinical management of patients.

## Author contributions

**Conceptualization:** Hongxia Yan.

**Data curation:** Hongxia Yan.

**Investigation:** Jianxin Zhang, Zhe Guan.

**Supervision:** Xinfeng Cai, Zhiying Hao.

**Validation:** Jianxin Zhang, Zhe Guan.

**Writing – original draft:** Hongxia Yan.

**Writing – review & editing:** Hongxia Yan.
